# Would compassion be able to intervene?

**DOI:** 10.1017/S1478951524001925

**Published:** 2025-01-24

**Authors:** Tadashi Nishimura, Hajime Fujimoto

**Affiliations:** 1 Department of Pulmonary Medicine, Mie Chuo Medical Center, Tsu, Japan; 2 Department of Pulmonary and Critical Care Medicine, Mie University Graduate School of Medicine, Tsu, Japan

To the editor,

As someone who has been involved in the treatment of lung cancer, I was impressed by the work in 2010 of Dr. Temel and colleagues that early palliative treatment improves overall survival (Temel et al. 2010). Most recently, Dr. Temel and colleagues’ approach of providing stepped palliative care was published (Temel et al. 2024). These trends indicate that palliative care remains important in lung cancer treatment. However, I guess that the problems in implementing these papers are the uneven distribution of palliative care specialists in different regions and the differences in individual physicians’ attitudes toward patients and palliative care.

The study by Andrea Bovero et al. is a great study that relates patient dignity and symptoms to the abstract concept of physician compassion (Bovero et al. 2024). I try to be a good clinician to my patients, and this is an excellent study that shows what that is and what behaviors are important to them. However, as the authors mention, there are some limitations to this study. It is a cross-sectional study and only healthcare providers willing to participate were selected, and these could be biases. Most shockingly, The compassion patient survey (COMP_PT)was associated with the Patient Dignity Inventory (PDI), whereas the compassion healthcare provider survey (COMP_HCP) was not. Does this mean that it is difficult to intervene in the PDI by educating and raising awareness among health care providers? And are there currently intervention studies underway or planned to address these? I intend to personally instruct other health care providers on the content of the COMP_PT and HCP from this study, but I would like to hear the authors’ next steps. If these results are to be used in an intervention study, I guess that a prospective study is needed to compare the results of the introduction of education at different facilities and to compare patient dignity and symptoms over time.

Of the 2 problems indicated in the first paragraph, the problem of uneven distribution of palliative care physicians has begun to show evidence of using remote technology (Greer et al. 2024). In order to provide optimal palliative care to more patients, I hope that the study by Andrea Bovero et al. and the education using it will eliminate the differences in awareness among individual physicians.
